# Antidiabetic and Toxicological Effects of the Tea Infusion of Summer Collection from *Annona cherimola* Miller Leaves

**DOI:** 10.3390/plants11233224

**Published:** 2022-11-24

**Authors:** Jesús Martínez-Solís, Fernando Calzada, Elizabeth Barbosa, Juan Manuel Gutiérrez-Meza

**Affiliations:** 1 Sección de Estudios de Posgrado e Investigación, Escuela Superior de Medicina (ESM), Instituto Politécnico Nacional, Plan de San Luis y Salvador Díaz Mirón S/N, Col. Casco de Santo Tomás, Mexico City CP 11340, Mexico; 2 Unidad de Investigación Médica en Farmacología, UMAE Hospital de Especialidades 2° Piso CORSE Centro Médico Nacional Siglo XXI, Instituto Mexicano del Seguro Social, Av. Cuauhtémoc 330, Col. Doctores, Mexico City CP 06720, Mexico

**Keywords:** *Annona cherimola* Miller, tea infusion, antihyperglycemic activity, subchronic oral toxicity, HPLC-DAD analysis, flavonoid glycosides

## Abstract

*Annona cherimola* Miller (**Ac**) is a plant used in Mexican traditional medicine for the treatment of diabetes. In this work, the tea infusion extracts obtained from 1.5 g of leaf powder from **Ac** collected in May (AcMa), June (AcJun), July (AcJul), and August (AcAu) were evaluated on streptozocin-induced diabetic (STID) mice and for subchronic toxicity in STID and non-diabetic (ND) mice. In addition, extracts were subjected to high-performance liquid chromatography with diode array detection (HPLC-DAD). Results showed that the tea infusion extract of the sample collected in August (AcAu) exhibited the most significant antihyperglycemic activity during all acute assays. The analysis of the extracts (AcMa, AcJu, AcJul, and AcAu) by HPLC-DAD revealed that flavonoid glycosides, rutin, narcissin, and nicotiflorin were the major components. In addition, the sample AcAu contained the best concentration of flavonoids. In the case of subchronic oral toxicity, the AcAu sample did not cause mortality in STID mice, and histopathological analysis revealed significant improvement in the changes associated with diabetes in the liver and kidneys. These findings suggest that the **Ac** leaves collected in August may be a source of flavonoids such as rutin, with antidiabetic potential. In addition, these findings support the use of **Ac** to treat diabetes in traditional medicine.

## 1. Introduction

Diabetes mellitus (DM) is a multifactorial disease characterized by a hyperglycemic chronic state that results from resistance to or lack of insulin secretion from the pancreas [1]. Particularly, DM type 2 is the most frequent, representing the largest proportion of all diabetes and affecting over 537 million people worldwide in 2021 [2]. This disease is commonly related to pathological changes in the structure and function of several organs such as the liver, as well as microvascular progressive deterioration, which is characteristic of diabetic nephropathy [3,4]. These changes are consequences at least in part to disturbances in carbohydrates and lipid metabolism derived from chronic hyperglycemia [5] that leads to the synthesis of advanced glycation end products (AGEP) and are related to oxidative stress due to the increase in releasing free radicals and lipid peroxidation, as well as attenuating the antioxidant protection as a result of non-enzymatic glycosylation of circulating and intracellular proteins [6], resulting in harmful damage mainly to the liver, kidney, and other tissues [7,8].

Currently, there are different kinds of drugs with anti-diabetic effects that improve glucose uptake or avoid blood glucose rise [9,10]. However, all these drugs induce side effects as a result of their continuous use, generating the need to search for other complementary treatments to prevent the early appearance of microvascular complications and damage in organs or to stop its progression [11]. One of the main sources of novel antidiabetic complementary treatments are medicinal plants that have been used by traditional medicinal practitioners around the world [12]. In industrialized regions, over 50% of the population look up to traditional medicine and herbal remedies to attend to health care and also have used complementary or alternative medicine at least once [11]. Moreover, the addition of herbal teas to oral hypoglycemic therapy has shown a potent synergistic effect in hyperlipidemia and DM management [4]. Unfortunately, although studies have shown the positive effects of plant extracts [13], it is still necessary to know how the environmental conditions affect the particular components and activities of the extracts [14] in order to validate the effect of these products. Therefore, more studies are necessary to evaluate the association between phytochemical variations and biological and toxicological effects in preclinical models in order to propose their possible use in humans.

*Annona cherimola* Miller (**Ac**) belongs to the *Annonaceae* family [15]. It is an evergreen tree of edible fruit known as Annona or cherimoya by local populations [16] and is distributed in subtropical areas in America and also around the world [17]. It is from 3 to 10 m tall, often low-branched, and somewhat shrubby or spreading. The semi-deciduous leaves are ovate-lanceolate, sometimes obovate or elliptical, 12–20 cm × 8 cm, and persistently have brownish-velvety tomentose underneath. They are alternate and two-ranked, with minutely hairy petioles, slightly hairy on the upper surface and velvety on the underside. Flowers are fragrant and extra-axillary, often opposite a leaf at the base of a branchlet and usually solitary with three outer oblong-linear tepals, up to 3 cm long, greenish to pale yellow, marked with a purple spot at the base inside; with three very small inner tepals, reddish to purplish; androecium consisting of numerous free fleshy stamens, spirally arranged on the basal part of a conical receptacle; gynoecium comprising numerous free pistils on the upper part of the receptacle. **Ac** is known to be eaten as a food and used in folkloric medicine [18]. Local populations use this plant for the treatment of diseases such as gastrointestinal disorders, worms, and diarrhea, as well as diabetes [19]. Phenolic and aromatic compounds such as rutin as well as other flavonoid glycosides are highlighted due to having been identified to be present in the most amount in the leaves of **Ac** [20] and to be responsible for a wide number of biological effects [21], including having antidepressant [22], pro-apoptotic [23], antilipidemic [24], and antihyperglycemic properties [25]. Although its leaves are present almost all year round, the changes in its antihyperglycemic and toxicological effects as a consequence of its appearance, as well as the environmental conditions and the number of active compounds in the aqueous extract of this species, are unknown.

Therefore, our study aimed to evaluate the effect of the tea infusion extract of **Ac** (IELAc) leaves from serial collections and the histopathological analysis of liver and kidney samples in streptozocin-induced diabetic (STID) and non-diabetic (ND) mice to obtain information on the best time to use it as a possible new adjuvant safety treatment for diabetes mellitus.

## 2. Results

### 2.1. Effect of the Tea Infusion Extract from A. cherimola Miller in STID Mice

To evaluate the effect of IELAc obtained from the different collections, we administrated it in streptozocin-induced diabetic (STID) mice. The blood glucose level of the treated groups was measured after treatment at 0, 1, 3, 5, and 7 h post-administration. As observed in Table 1, the single administration of the tea infusion extract made from the collections in May (AcMa), June (AcJun), and July (AcJul) caused a significant reduction of the hyperglycemic values in STID mice from the first to the seventh hour after the administration compared with the STID control groups. Nevertheless, the antihyperglycemic effect was higher with the IELAc made from the collection of the August (AcAu) control groups. Moreover, in the case of the acarbose group, the antihyperglycemic effect was observed only from the first to the fifth hour post-administration, rising to hyperglycemic levels at the end of the assay and being significantly lower than the effect observed with the IELAc-treated groups. Finally, the non-diabetic (ND) control group showed normoglycemic values throughout the evaluation and no mortality was observed in any case

### 2.2. HPLC-DAD Analysis of the Tea Infusion Extract from Annona cherimola (IELAc)

**Figure 1:**
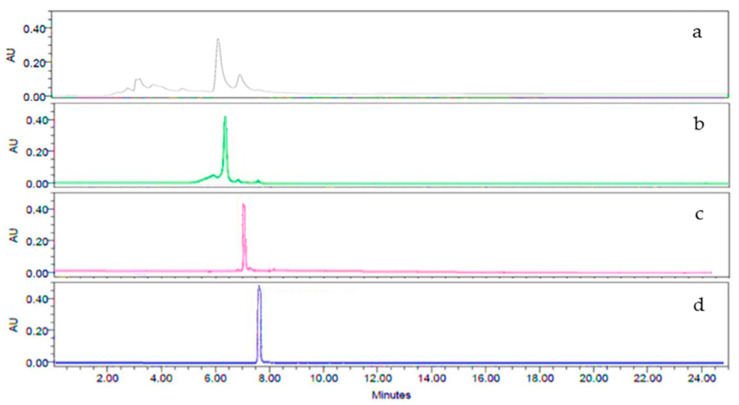
Representative high-performance liquid chromatography with diode array detection (HPLC-DAD) analysis at 254 nm of a sample of tea infusion extract of the leaves from **Ac** (IELAc) (**a**) and the flavonoid standards used rutin (**b**), narcissin (**c**), and nicotiflorin (**d**).

The analysis by HPLC-DAD of three samples of each tea infusion extract from **Ac** was performed to evaluate the tea infusion composition. As can be observed in Figure 1, the presence of constituents with retention times ranging between 4.0 and 8.0 min was confirmed. These peaks were compared using the standards of the flavonol glycosides corresponding with rutin, narcissin, and nicotiflorin. Due to rutin being the highest compound found, it was used as a marker and measured through the collection period to evaluate the difference in the collections.

**Figure 2:**
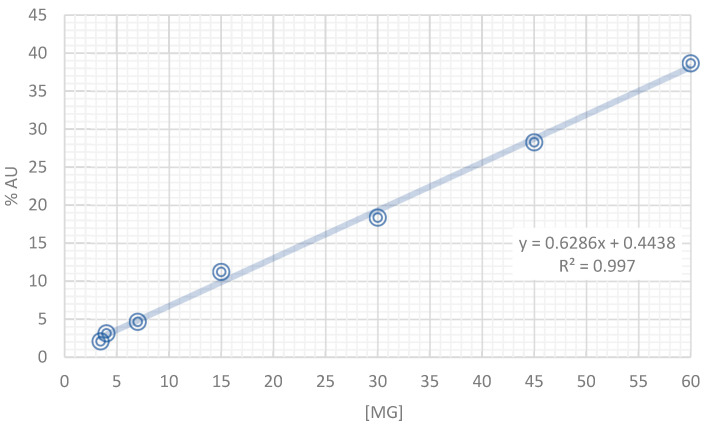
Calibration curve of the standard rutin (the major compound in the tea infusion extracts of **Ac**) of high-performance liquid chromatography with diode array detection (HPLC-DAD) analysis at 254 nm.

The calibration curve of rutin—the major flavonoid glycoside—into the extract at 254 nm is shown in Figure 2. In addition, the concentration of rutin, narcissin, and nicotiflorin throughout the collection period (May (AcMa), Jun (AcJun), Jul (AcJul), and August (AcAu) obtained quantitatively through the %AU is shown in Table 2. The tea infusion extract was made with 1.5 g of fresh-harvested leaf powder from **Ac** from different months. Three samples of each tea infusion extract were analyzed by HPLC-DAD to determine the changes in the concentration of these flavonoids. As can be observed, the yield and concentration of these flavonoids were increasing, being significantly greater with the infusion made with the leaf powder collected in August (AcAu). Therefore, this data reflects that collecting the plant material in August is the best moment compared with the previous months. Moreover, an infusion extract from the plant material in this month could exert the best effect due to the high concentration of its active compounds.

### 2.3. Histological Results

#### 2.3.1. Histological Changes on the Liver of Non-Diabetic (ND) and STID Mice Treated with the Tea Infusion Extract of Ac

**Figure 3:**
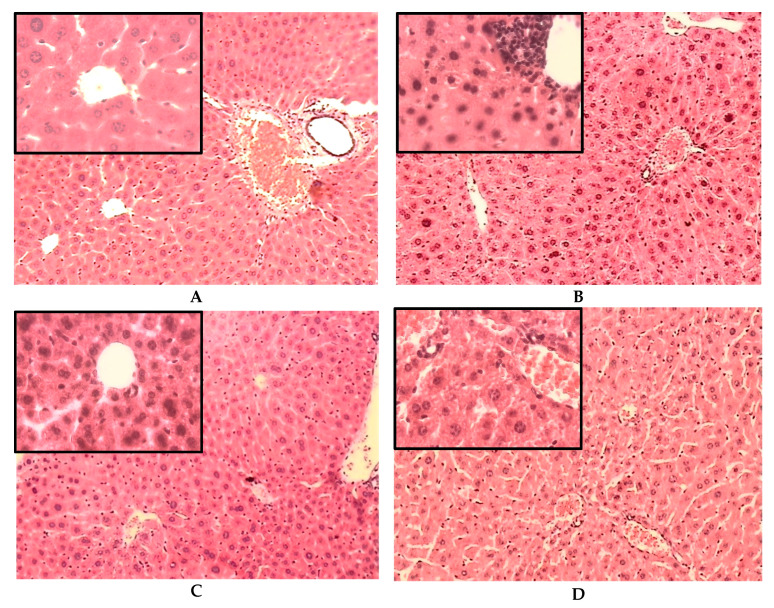

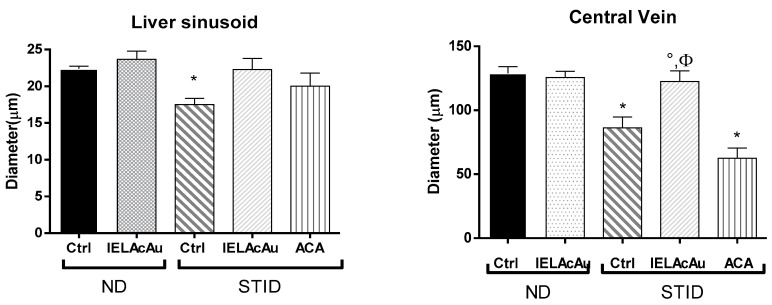
(**Top**) Representative photomicrographs with H&E staining of transversal liver sections after the administration for 28 days of IELAcAu in non-diabetic (ND) and streptozocin-induced diabetic (STID) mice. (**A**) ND control. (**B**) STID control. (**C**) STID with IELAcAu (300 mg/kg bw). (**D**) STID with acarbose (ACA) (50 mg/kg bw) (magnification 10× and box 40×). (**Bottom**) Quantitative analysis of vascular diameter and sinusoids of liver sections. Results are expressed as the mean ± SEM, *n* = at least 9 fields in each group. * *p* < 0.05 vs. ND control; ° *p* < 0.05 vs. STID control; ^Φ^ *p* < 0.05 vs. acarbose.

The analysis of the photomicrographs of liver samples stained with hematoxylin and eosin (H&E) of all groups is shown in Figure 3. The architecture of hepatic tissue of ND control mice (Figure 3A) was observed with polygonal hepatocytes with central euchromatic nuclei and eosinophilic granular cytoplasm. Some hepatocytes were binucleated. Sinusoids were found converging towards the central vein. Portal areas contained connective tissue and portal triad. The architecture of the liver sinusoids and the diameter of the central vein were normal. In addition, some Kupffer cells were in the sinusoidal space, as well as some endothelial cells lining the central vein. After the treatment with IELAc from August in ND mice, no changes were found in the liver histology, which appeared more or less like the control group and preserved the overall structure of the hepatic lobe.

The representative photomicrograph of the liver sections of the STID control group (Figure 3B) revealed a reduction in the diameter of the central veins and blood sinusoids, which also showed as less arrayed. Moreover, cellular damage characterized by ballooned hepatocytes with vacuolated cytoplasm was found. Signs of apoptosis as karyorrhexis, fragmented nuclei, altered cytoplasmic regions, as well as accentuated perivascular inflammatory congestion were also observed. Some hepatocytes appeared with a small cytoplasm and big dark nuclei. These changes observed in the liver samples of the STID control mice were a probable consequence of fat metabolism disorders associated with diabetes induced by streptozocin administration.

The representative image of the liver sections of treatment with IELAc from August in STID mice (Figure 3C) appeared somewhat more like the normal histological architecture. The hepatocyte appearance was observed as more arrayed. Less vacuolization was also observed in the cytoplasm of the hepatocytes. Sinusoid cordons were observed as more linear, with discrete inflammatory processes. Finally, the vein and sinusoid diameter were like the ND control group.

In the case of the liver sections of the diabetic mice treated with acarbose (Figure 3D), a diffuse inflammatory process was observed compared with the STID control group. Moreover, the cordons of the hepatocytes were found disarrayed and ballooned, with a great number of intracytoplasmic vacuoles; this is maybe associated with fatty liver conditions in diabetes. The diameter of the veins, but not of the liver sinusoids, was reduced.

#### 2.3.2. Histological Changes on the Kidneys of Non-Diabetic (ND) and STID Mice Treated with the Tea Infusion Extract of Ac (IELAc)

**Figure 4:**
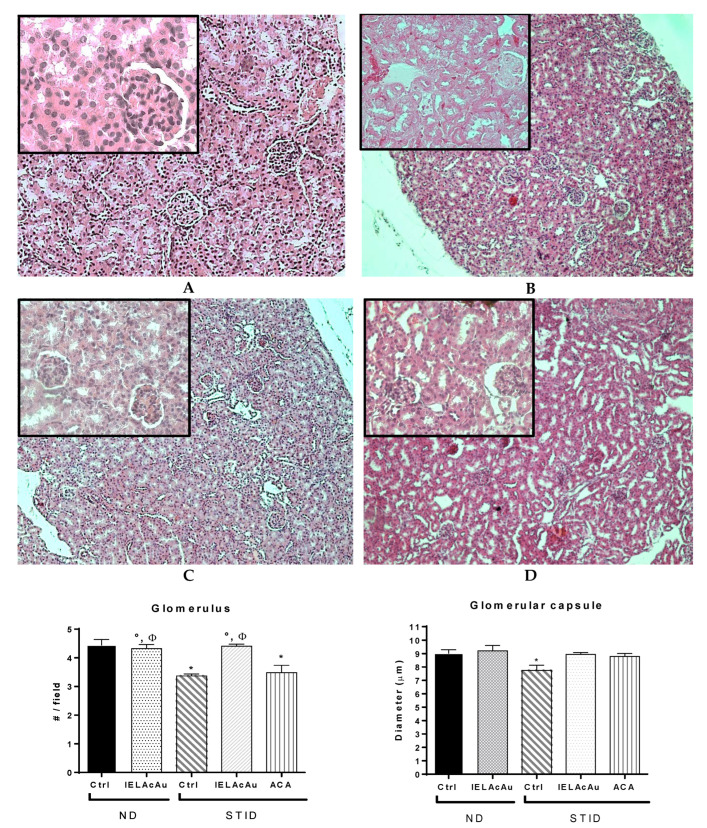

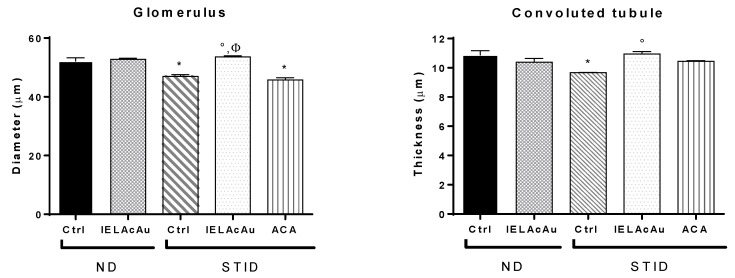
(**Top**) Representative photomicrographs with H&E staining of transversal kidney sections after the administration of IELAcAu in non-diabetic (ND) and streptozocin-induced diabetic (STID) mice. (**A**) ND control. (**B**) STID control. (**C**) STID with IELAcAu (300 mg/kg bw). (**D**) STID with Acarbose (ACA) (50 mg/Kg bw) (magnification 10× and box 40×). (**Bottom**) Quantitative analysis of glomerulus and convoluted tubules of kidney sections. Results are expressed as the mean ± SEM, *n* = at least 9 fields in each group. * *p* < 0.05 vs. ND control; ° *p* < 0.05 vs. STID control; ^Φ^ *p* < 0.05 vs. acarbose.

The photomicrographs of the histological kidney samples stained with hematoxylin and eosin (H&E) of all experimental groups (Figure 4) were analyzed to explore the effects of the administration of the tea infusion extract from August (IELAcAu) on the renal architecture in ND and STID mice. In the case of the ND control group (Figure 4A), the glomerular structure was intact, and the macula cells were observed with adequate size and disposition, with a normal proportion of glomerular sacs per field. Moreover, the renal tubules were arranged homogeneously with cubical epithelial cells with central homogeneous nuclei and their brush border, without signs of atrophy or cytoplasmic vacuolar lesion. Additionally, there was no inflammatory infiltration in the interstitium and no signs of pathology or hemorrhagic areas in the cortex or medulla. The treatment with IELAcAu in the ND mice did not cause observable or significant changes in the renal architecture compared with the ND control mice.

In the case of the representative photomicrographs of the STID control group (Figure 4B), a significant diminution in the number of the glomerulus was observed. In addition, the renal tubules were arranged disorderly and inflammatory cells as well as interstitial infiltration and extracellular matrix deposits were observed, suggesting a fibrosis process. Moreover, atrophy signs such as glomerular contraction and reduced diameter as well as loss of continuity of the convoluted tubules were also observed.

The representative photomicrographs of the renal samples of the STID mice treated with IELAcAu (Figure 4C) were found with fewer glomerular lesions compared with the STID control group. In addition, the glomeruli were less contracted, and the glomerular diameter was recovered. Tubular distribution was more or less like the ND control groups, without signs of atrophy, inflammatory infiltration, or hemorrhagic areas.

However, in the STID group treated with acarbose (Figure 4D), kidney sections showed glomeruli significantly contracted with reduced diameter, suggesting signs of renal dysfunction. In addition, convoluted tubules were disordered, with loss of continuity in some regions; however, less inflammatory infiltration was found compared with the STID control group.

## 3. Discussion

Diabetes mellitus management is an important scope in public health [26], but, although diabetes cannot be cured, diabetic complications can be managed successfully by efficiently normalizing the glucose and lipid blood levels [27]. Many attempts have been used to treat DM, but objectives have not yet been achieved due to several limitations of the current therapies [3]. This leads to the need to find complementary treatments that must be effective without undesirable side effects [26]. In this sense, people who usually drink herbal teas are less likely to develop diabetic disorders [28]. In addition, medicinal plants are known for their antidiabetic properties [29] and are used as supplements to help achieve glycemic control in people with diabetes. Furthermore, the consumption of natural antioxidants derived from herbal extracts has increased recently due to their specific biological activity and low toxicity, making them potential compounds for the prevention of oxidative stress disorders that occur in metabolic syndrome and diabetes [30]. Some antioxidant flavonoids are considered dietary supplements in the prevention or treatment of diabetic complications [27], with no reported toxicity or side effects compared with current hypoglycemic therapies that have inevitable side effects [31].

Medicinal plants have been used in folkloric medicine for different purposes [32]. In this regard, the genus *Annona* includes several species that have been studied due to their properties to treat illnesses [33]; therefore, it is a good source to search for compounds with medicinal properties. The aqueous extracts of *Annona muricata*, *A. stenophylla*, and *A. macroprophyllata* have shown antidiabetic, antioxidant, antilipidemic, and antinociceptive effects that are attributed to the presence of phenolic compounds in their leaves [34,35,36,37]. The antidiabetic properties of extracts from **Ac** have also been studied in previous publications [25] and it has been established that making a tea infusion from the leaf powder is a good way to produce the antilipidemic effect [38]; therefore, this extract was used in the present study. Nevertheless, no reports showing the histological changes induced by the treatment with this extract or changes in the effect related to the harvest period of the leaves have been studied.

First, the analysis by HPLC-DAD of the samples of each tea infusion extract made with 1.5 g of leaf powder from the seriated harvest showed the presence of compounds in which the glycoside flavonoid rutin was highest, followed by narcissin and nicotiflorin. The quantification of rutin was considered a marker compound of the tea infusion extracts due to the major compound with demonstrated activity [39]. Flavonoids are a class of naturally occurring benzo-γ-pyrone derivatives of plant origin [27], widely distributed and found in many vegetal species, and have been used as a restorative in preparations of herbal medicine with a non-toxic and protective behavior [40]. Furthermore, flavonoids have been studied for the redox effect associated with their phenolic groups and thus are proposed as effective supplements against oxidative stress-related secondary complications [41]. Moreover, flavonoids exert antidiabetic and antilipidemic effects, suppressing the intestinal absorption of glucose, cholesterol, and triglycerides by the inhibition of *α*-glucosidase activity [42], stimulating the glucose transporter 4 (GLUT4)-mediated glucose uptake, increasing the insulin sensibility [43], and suppressing the pancreatic phospholipase-A2 (PA2) essential for lipid hydrolysis [44]. These concentration-related activities are considered important for preventing a wide variety of diseases, including diabetes and other related illnesses such as inflammation and hepatic damage [27]. Particularly, it has already been demonstrated that the extract from **Ac** reduces cholesterol absorption in intestinal cells [24], has an inhibiting *α*-glucosidase activity that is responsible for its antihyperglycemic effect [25], and its infusions reduce the blood level of cholesterol and triglycerides in mice [38]; therefore, this shows that the aqueous extract could have beneficial effects over other diabetic related complications.

The evaluation of the seriated collections shows that the growth state of the leaves changes the flavonoid concentration; this can modify the antidiabetic effect exerted, being higher in the months when the growth is accentuated. In this sense, phytochemical analysis in other studies has demonstrated that the therapeutic effect and composition of extracts vary greatly depending on the site or season of the collection, even in the same country or in consecutive months [11,45]. Although **Ac** is considered an evergreen species due to the presence of leaves during almost the whole year, the season could modify the number of metabolites in its extracts [46]. Therefore, in this work, we consider it important to evaluate the effect of the samples collected at serial times to determine whether these environmental changes are capable of inducing modifications in the reported beneficial effects. We analyzed the growth conditions of the specimen, the appearance of the leaf, and how this characteristic can modify the number of active compounds, resulting in a variation in efficacy. Our results, based on the measuring of flavonoids in the tea infusion extract from **Ac** by HPLC-DAD, showed that the content of the major marker flavonoid rutin, as well other flavonoids that are implied at least partially in the therapeutic effect, changes throughout the annual course, being higher during the rainy season months, leading to modifications in the potency of the antihyperglycemic effect. This makes sense, since the active compounds are highly polar, and maybe the metabolic pathway could be influenced by fluvial conditions in that season. Although this species sheds its leaves in the last of winter and the beginning of spring, the growth occurs during the annual rainfall season [45] and is related to the pollination and flowering period [47,48,49], which, in subtropical places in America, takes place mainly from the second half of May to August. Moreover, it is known that the best growth of this species occurs under high relative humidity and vapor pressure [50,51], not doing so well at high temperatures or high drought or CO_2_ amounts [52,53]. This information indicates that environmental conditions could influence not only the leaf appearance of this species but could have an impact on the concentration of bioactive compounds and, later, on the biological activity. Therefore, collecting fresh and mature leaves during the rainy season in August is the best time to obtain a great proportion of active compounds and good activity.

On the other hand, chronic hyperglycemia in DM is related not only to vascular damage that increases the cardiovascular risk but also to changes in the architecture of several organs, resulting in their dysfunction. The impaired function of the liver and the kidneys is the most common complication of diabetes [4]. In this work, we wanted to analyze whether the treatment with IELAcAu would be able to induce beneficial changes in both these organs in STID mice.

The liver is the major organ involved in the detoxifying process [54] and plays an important role in the metabolism of carbohydrates, lipids, and other substances [27]. For this reason, it is one of the organs most susceptible to oxidative stress in diabetes and is affected by insulin resistance [14], which stimulates the release of free fatty acids from the adipose tissue and its accumulation inside the hepatocytes [55]. This lipid imbalance related to hyperglycemia has been associated with hepatic damage [56,57] and results in a pathological condition known as non-alcoholic fatty liver disease (NAFLD) or steatohepatitis [4]. Moreover, the excess lipid infiltration and triglyceride accumulation in the liver cells release pro-inflammatory cytokines and generate reactive oxygen species (ROS) [58]; this plays a pivotal role in the pathophysiology of diabetic hepatotoxicity [27]. Thus, the search for alternative treatments must be oriented not only toward improving hyperglycemia but also to reducing the pathological changes associated with diabetes in the liver architecture that is responsible, at least in part, for the chronic failure of the treatment. Previously, it has been demonstrated that the extract of **Ac** can improve the lipid profile in mice [38] by reducing the blood levels of cholesterol and triglycerides and by increasing the HDL level. On the other hand, it is already known that rutin inhibits cholesterol absorption from the intestine, thereby lowering the serum and hepatic lipid content [56]. This effect protects the liver from pathological changes associated with hyperlipidemia caused by insulin resistance [59]. Moreover, flavonoids reduce the synthesis of LDL, blocking the inflammation in the liver [56]. Therefore, the presence of rutin as the major compound in the infusion extract explains its antihyperlipidemic effect, which is reflected in the reduced vacuolization of the liver sinusoids and less inflammatory proliferation near the central veins of the liver sections samples in the STID mice group treated with IELAcAu in this work, keeping the architecture more like the ND mice. This is in accordance with other studies that have shown that dietary intervention with flavonoids reduces lipid peroxidation and histopathological changes in the liver of diabetic mice [27]. Moreover, the administration of IELAcAu in the ND mice did not cause appreciable or quantitative changes in the liver, which reflects that this treatment does not lead to pathological modifications, and it is safe in this model.

Diabetic nephropathy (DN) is another of the main microvascular long-term complications related to chronic hyperglycemia and causes end-stage renal failure [4,60], leading these patients to the need for peritoneal dialysis and hemodialysis [11]. This disease involves factors such as the overproduction of reactive oxygen species (ROS) and inflammatory infiltration that changes the renal parenchyma [61]. In diabetes, the accumulation of high-glucose amounts leads to an improper renal function [62] due to the overproduction of AGEP, which plays an important role in the fast progression of DN [63,64]. AGEP induces ROS production and promotes inflammatory infiltration [63], generating glomerular and tubule-interstitial damage [65]. In this work, the analysis of kidney section samples in the STID control group revelated changes in the renal glomerulus and tubules associated with DN. Nevertheless, the treatment with the IELAc significantly reduced the changes associated with DN. Previous studies have reported that flavonoids such as rutin have strong inhibitory activities against AGEP formation [66] and ameliorate renal function by decreasing the expression of inflammatory cytokines involved in the progression of DN [11]. Similarly, herbal teas have shown the potential to ameliorate diabetic renal dysfunction thorough inhibiting AGEP formation and cutting off inflammatory pathways via trapping metabolite methylglyoxal [4]. In this sense, it has been shown that the IELAc is also able to reduce the HbA1c levels in STID mice [38]. HbA1c is the main AGEP used as a marker to evaluate the progression in people with diabetes [67]. In the present work, the reduction of the changes associated with DN observed in the STID mice group treated with IELAc could be explained at least in part by the inhibitory α-glucosidase activity of this infusion, which reduces the rise of the glucose level in the bloodstream, thus preventing its linkage with circulant or cellular proteins, and therefore the synthesis of AGEPs, resulting in the reduction of the renal glucotoxicity and DN.

Polar flavonoids in IELAc may be responsible for its protective effect observed against the damage of diabetic complications related to oxidative stress on the liver and kidney. Polyphenolic compounds as flavonoids have great potential in reducing oxidative stress [27] by lowering the malondialdehyde (MDA) [40], increasing glutathione and superoxide dismutase levels [68], and preventing lipid peroxidation [4], which is related to lipid synthesis. In this sense, the flavonol glycoside rutin is one of the major flavonoid compounds that occurs in food [56]. It has great antioxidant activity, realized through known mechanisms such as activating the intercellular antioxidant enzymes, scavenging oxygen radicals, protecting against lipid peroxidation, and chelating metal ions [27]. These antioxidant mechanisms seem to be primarily associated with their structural features as the presence of functional hydroxyl groups on the A-ring and B chain [14]. Moreover, it has already been observed that the intensity of this beneficial effect could be associated with the first-pass effect, where some supplements with antioxidant properties undergo biotransformation [27]. In the same line, it has been shown that flavonoids effectively avert morphological changes in the liver [56]. Other studies have demonstrated that the administration of the aqueous extracts from *Annona muricata* protects against damage induced by drugs or infections and can revert liver failure [69,70]. Our current results show that the tea infusion extract of **Ac** is also able to induce a reduction in the pathological changes of the hepatic and renal parenchyma; this may be influenced by the therapeutic antioxidant potential of the polyphenolic compounds present in the infusion extract and follows other studies that report that aqueous extracts rich in flavonoids exert an antidiabetic effect [71]. Therefore, this could explain other benefic effects over damage associated with diabetes and could provide evidence for future clinical testing.

Finally, taking into consideration the first results of the histological analysis of the treatment with the tea infusion extract of **Ac** in streptozocin-induced diabetic (STID) and non-diabetic (ND) mice, the findings suggest that the presence of polar flavonoids has a positive effect on the liver and kidney, while having no pathological effect on the normal architectures in ND mice. Moreover, the polyphenolic compounds present in the infusion extract allow it to be considered a dietary supplement that could contribute to the treatment of diabetic complications and other diseases related to oxidative stress in humans and could also lead to partially show the beneficial role of the infusion extract for other states of metabolic syndrome, such as dyslipidemia and non-alcoholic fatty liver disease (NAFLD), or for chronic complications such as diabetic nephropathy (DN). However, further research to evaluate the security of other possible effects of IELAc in other regulations of metabolic processes and pregnancy is currently underway. It is relevant to note that these data support the use of an infusion of **Ac** as it occurs in folkloric medicine that could have a protective effect on reducing the risk factors that contribute to the death of people with diabetes and could give further information on understanding the antihyperglycemic effect of this herbal formulation as a good agent to treat not only hyperglycemia but also several other complications related to diabetes mellitus.

## 4. Materials and Methods

### 4.1. Plant Material

For this study, *Annona cherimola* Miller (**Ac**) leaves were collected monthly from a specimen under organic conditions from July to August 2020 in the town of San Gregorio Atlapulco, Xochimilco, Mexico (19°15′08.3″ N 99°03′14.3″ W). Then, samples of every collection were authenticated by the botanist MSc Santiago Xolapa Molina of the Medicinal Plant Herbarium (IMSSM) in the Mexican Institute of Social Security (IMSS) and stored for further reference. The name of the plant was also verified at http://www.theplantlist.org (http://www.theplantlist.org/tpl1.1/record/kew-2640812 accessed on 17 November 2022). Finally, leaves were washed twice with purified fresh water and left to dry in the dark at room temperature. For use after that, they were finely powdered using a commercial blender and stored in air tight Ziplock bags for extract obtention and further analysis.

### 4.2. Reagents

Streptozocin (≥75% α-anomer basis, PN: S0130-5G), nicotinamide (≥99.5%, PN: 47865-U), acarbose (PN: PHR1253-500MG), (3-Aminopropyl) trimethoxysilane, buffer solution (citric acid/sodium hydroxide/hydrogen chloride, pH 4.00, CC: 109445), Harris hematoxylin (HH280) and eosin (I.C. 768) were purchased from Sigma-Aldrich^®^ (Sigma^®^, Saint Louis, MO, USA); saline solution 0.9% (solution 1000 mL) and DX-5 glucose solution 5% were purchased from PISA^®^ Pharmaceutics (PISA^®^, Mexico City, México); histological paraffin (fusion point 56–58 °C) was acquired from a commercial source. Ethanol, butanol, xylene, and solvents of analytical grade were used.

### 4.3. Plant Extract Procedure

For the elaboration of all the tea infusion extracts of **Ac** (IELAc) used in this work, 1.5 g of the powder obtained from the leaves of different collections was placed in a tea bag and soaked in 125 mL of water that was pre-boiled and distilled for 20 min to match traditional common usage. The resulting extract was then filtered through grade 1 filter paper and concentrated on a rotary evaporator (Büchi Labortechnik AG, Flawil, Switzerland) at 40 °C under reduced pressure. This process was carried out monthly with all the extracts. Finally, the sticky precipitate obtained was stored at 4 °C for further reference and an aliquot of the extract residue was weighed and resuspended in water for administration. In all cases, the extraction yield per gram of vegetable powder was evaluated by the difference in the weight of the container before and after pouring the extract.

### 4.4. Characterization of the Tea Infusion Extract of Leaves from Ac by High-Performance Liquid Chromatography

Once obtained, the infusion extracts of the leaves from **Ac** (IELAc) of three samples from each monthly extract were analyzed by an HPLC-diode array detection (DAD) to identify compounds in the extracts; the concentration of the main one was also measured throughout the entire collection period. For this, an HPLC-DAD Waters 2795 liquid chromatography system was used, coupled with a Waters 996 photodiode array detector and an analytical Millennium 3.1 workstation equipped with a C18 column (200 × 15 mm), the particle size of 5 μm (Spherisorb S50D52, Waters Corporation, Milford, MA, USA). For the analysis, 10 mg of IELAc was solved on 10 mL of ethanol and a sample volume of 20 μL from that solution was injected. For elution, a system comprising a binary mobile phase of acetonitrile solvent/acetic acid 2% in water (A) and acetonitrile 100% (B) was used. The chromatograph operating conditions were programmed to give the following gradients: 1st stage, linear gradient of 80 (A)/20 (B) for 8 min; 2nd stage, linear gradient of 40/60 for 5 min; 3rd stage, linear gradient of 30/70 for 6 min; 4th stage, linear gradient of 90/10 for 6 min with a flow rate of 1 mL/min of the mobile phase. The detection was carried out at room temperature and at a total elution time of 25 min at a wavelength (λ) of 254 nm, because the highest absorption peak of these flavonoids is found at this wavelength [45]. Then, the collected data were plotted. Reference standards of flavonoid rutin, narcissin, and nicotiflorin, as well as acetonitrile, ethanol, and acetic acid of HPLC grade, were acquired from Sigma (St. Louis, MO, USA) and were prepared and analyzed separately under the same conditions described above. In all cases, the water used was of HPLC quality and purified in a Milli-Q system (Millipore, Bedford, MA, USA). The presence of substances in the extract of the leaves from **Ac** was confirmed by comparing the retention times and the UV spectra of each of the standards. The concentration of flavonoid glycosides in each infusion extract was obtained by measuring the area under the curve (AU) of each compound identified in its obtained chromatogram.

### 4.5. Experimental Animals

For this study, healthy albino Balb/c mice of both sexes aged 8–10 weeks (25 ± 5 g) were obtained from the Animal House of the National Medical Center “Siglo XXI” from Instituto Mexicano del Seguro Social (IMSS), housed under standard laboratory conditions at a temperature of 22 °C ± 2 °C, 50% of humidity with a 12-h light/dark cycle, and fed with a standard diet and purified water *ad libitum*. This study was approved by the Specialty Hospital Ethical Committee of the National Medical Center “Siglo XXI” from IMSS, with the registered numbers: R-2020-3601-038 and R-2019-3601-004. Furthermore, all the experimental animal models were performed following the Mexican Official Regulations on animal care and experimental management [72].

#### 4.5.1. Grouping

Animals were assigned randomly to different groups of six mice each, as follows: Groups 1 and 2 were assigned as non-diabetic (ND) and Group 2 was treated with IELAc at 300 mg/kg body weight (bw). Groups 3 to 5 were streptozocin-induced diabetic (STID) mice. IELAc at 300 mg/kg bw was given to Group 4 of STID mice. Group 5 received acarbose at 50 mg/kg bw, which was used as a reference drug. Groups 1 and 3 performed as a control and non-received treatment. All treatments were administrated by the oral route through a gavage and solved in distilled water; the volume was calculated according to the OECD guidelines [73] as 2 mL/100 g bw.

#### 4.5.2. Induction of Experimental Type 2 Diabetes

In the case of the STID model, the animals fasted previously overnight and experimental type 2 diabetes was induced by an intraperitoneal injection of freshly prepared STZ (100 mg/kg bw) solved in 0.1 M citrate buffer (pH 4.5). After 30 min, a single intraperitoneal dose of nicotinamide (240 mg/kg) in a saline solution of 0.9% was given. Then, the animals were allowed to drink a 5% glucose solution overnight to stabilize the drug-induced hyperglycemia [74]. To confirm an experimental type 2 diabetes model, a single dose of 5 mg/kg bw glibenclamide—a secretagogue drug—was given orally to the STID mice to confirm that the endocrine function of the pancreas was preserved and not completely obliterated due to the action of the STZ [75]. Glycemia was determined after the last administration, using the glucose oxidase method (Accu-Chek^®^ Performa Glucometer, Boehringer Mannheim, Germany) [76], using blood samples collected during the assay by puncture from the tail vein. Those animals with blood glucose values above 290 mg/dL and below 350 mg/dL on the third day after the STZ injection were considered diabetic and used in this study, while animals with capillary glycemia under these values were considered non-diabetic (ND).

### 4.6. Study of Variation of the Antihyperglycemic Effect of the Infusion Extract of Ac

To evaluate the possible changes in the antihyperglycemic effect induced by the IELAc made from seriated collections, the extract was administrated to streptozocin-induced diabetic (STID) and non-diabetic (ND) mice. Both control groups received water, while the experimental group received the IELAc from May (AcMa), June (AcJun), July (AcJul), or August (AcAu) at 300 mg/kg bw, given as described above. In this case, the blood glucose levels were measured at 0, 1, 3, 5, and 7 h after treatment, according to the method mentioned above.

### 4.7. Evaluation of the Sub-Chronic Effect of the IELAc

Once determined which extract exerts the greatest effect, we evaluated the sub-chronic assay at 300 mg/kg or acarbose 50 mg/Kg bw in STID and ND mice daily for 28 days. The animals were weighed weekly and kept in conditioned cages under standard conditions with free availability of food and water; changes in their behavior or physical appearance were recorded throughout the treatment.

### 4.8. Effect of the Treatment on Internal Organs and Histology

After the sub-chronic assay, the animals were weighed and euthanized according to animal management standards [72] by abdominal incision. Then, the abdominal visceral organs were excised, washed with physiological saline solution, and weighed to obtain the absolute weights. Later, they were fixed in 4% PFH and washed with bidistilled water. Before inclusion, different transversal portions of the liver and kidney were sectioned and dehydrated in a battery of alcohols of increasing concentrations and included in paraffin for further analysis. The paraffin blocks were sectioned with a microtome (Leica RM 2125RT) to obtain serial sections of 7 µm thickness. In total, 3 sections were obtained from each sample, with a separation of 100 µm from each other. Finally, the samples were stained with H&E [77]. For each histological sample, at least 9 fields were observed. Histological analysis and photographic recording were performed using an optical microscope Nikon Eclipse E600 (Nikon Instruments Inc., Melville, NY, USA) with a color video camera digital camera SONY Exwave HAD (Sony, Electronics Inc., Paramus, NJ, USA).

#### Quantitative Morphometric Measurement

To obtain morphometric data in this study, Image Pro Plus 5.1.0 Image Analysis and Processing Software was used (Media cybernetics, Rockville, MD, USA, EU). Nine non-overlapping fields in stained slides of each animal in each group were examined. Measurements were performed on 7 µm thick H&E-stained sections at a magnification of 10× and 40× to estimate:

1. Diameter of liver sinusoids in the control and the treated groups.
2. Diameter of central veins in the liver of the control and the treated groups.
3. Number of renal glomeruli per field observed in the control and the treated groups.
4. Diameter of the glomerular capsule in the control and the treated groups.
5. Diameter of the renal glomerulus in the control and the treated groups.
6. Diameter of convoluted tubules in the control and the treated groups.

### 4.9. Statistical Analysis

Data are expressed as the mean ± standard error mean (SEM). Statistical significance was determined by a one-way analysis of variance (ANOVA) followed by a Tukey test for multiple comparisons (GraphPad Prism Version 6.01; GraphPad Software Inc., La Jolla, CA, USA), considering the *p*-value < 0.05 as indicative of the significant difference between group means.

## 5. Conclusions

In this study, the therapeutic effect attributed to polar flavonoids in the tea infusion extract made with 1.5 g of *Annona cherimola* Miller leaf powder changes throughout the month of harvest in the summer season of the year. The sub-chronic administration of the extract made from leaves collected in August (IELAcAu) with the highest amount of flavonoids reduces the pathological changes in the liver and the kidney in STZ-induced diabetic (STID) mice, with no pathological changes observed in non-diabetic (ND) mice. These results support the use of an infusion extract of the leaves from **Ac** as a safe supplement useful for the treatment of diabetes mellitus complications.

## Figures and Tables

**Table 1:** Blood glucose levels of streptozocin-induced diabetic (STID) mice and nondiabetic mice (ND) at 0, 1, 3, 5, and 7 h post-administration of IELAc from May (AcMa), June (AcJun), July (AcJul), and August (AcAu).

Group	Blood Glucose Levels (mg/dL)				
	0 h	1 h	3 h	5 h	7 h
ND Control	135.3 ± 3.04 ^b^	140.3 ± 3.18 ^b^	127.4 ± 5.31 ^b^	140.6 ± 8.70 ^b^	122.0 ± 5.94 ^b^
AcMa	335.6 ± 5.95 ^a^	314.2 ± 2.44 ^a,b,c^	301.6 ± 5.67 ^a,b^	308.6 ± 2.95 ^a,b^	314.3.6 ± 8.38 ^a,b,c^
AcJun	307.5 ± 3.50 ^a^	253.0 ± 8.12 ^a,b^	269.1 ± 1.84 ^a,b,c,d^	295.6 ± 1.14 ^a,b^	318.3 ± 4.09 ^a,b,c^
AcJul	319.2 ± 6.71 ^a^	324.3 ± 7.55 ^a,b^	202.5 ± 9.75 ^a,b,c,d^	312.1 ± 5.96 ^a,b^	326.3 ± 10.45 ^a,b,c^
AcAu	325.5 ± 3.90 ^a^	283.0 ± 8.35 ^a,b,d^	245.8 ± 6.59 ^a,b,c,d^	258.8 ± 7.87 ^a,b^	210.0 ± 6.68 ^a,b,c^
ACA	343.5 ± 1.36 ^a^	301.0 ± 3.42 ^a,b^	314.5 ± 9.34 ^a,b^	295.0 ± 8.37 ^a,b^	366.0 ± 2.01 ^a^
STID control	343.3 ± 2.50 ^a^	391.6 ± 1.65 ^a,c^	379.0 ± 9.33 ^a,c^	375.5 ± 10.38 ^a,c^	386.0 ± 2.15 ^a^

The data are expressed as the mean ± SEM (*n* = 6) ^a^ *p* < 0.05 vs. ND control group; ^b^ *p* < 0.05 vs. STID control group; ^c^ *p* < 0.05 vs. ACA control group; ^d^ *p* < 0.05 vs. initial value. ND—non-diabetic mice; STID—STZ-induced diabetic mice; Aca—group administered acarbose 50 mg/kg as pharmacological control.

**Table 2:** The concentration of flavonoids in the tea infusion extracts of **Ac** throughout of the summer collection.

MONTH	Yield (mg)	(Rutin) (mg/g)	(Narcissin) (mg/g)	(Nicotiflorin) (mg/g)
AcMa	35.67 ± 0.18 ^c,d^	3.44 ± 0.17 ^d^	0.58 ± 0.02 ^c,d^	0.08 ± 0.002 ^c,d^
AcJun	49.24 ± 3.94 ^d^	4.30 ± 0.21 ^d^	1.38 ± 0.42 ^d^	0.31 ± 0.01 ^d^
AcJul	89.81 ± 8.03 ^a^	4.61 ± 0.43 ^d^	2.68 ± 0.89 ^a,d^	0.34 ± 0.07 ^a,d^
AcAu	93.17 ± 17.18 ^a,b^	7.96 ± 0.89 ^a,b,c^	4.24 ± 0.68 ^a,b,c^	0.52 ± 0.02 ^a,b,c^

The data are expressed as the mean ± SEM. *n* = 3 analyzed samples. ^a^ *p* < 0.05 vs. AcMa; ^b^ *p* < 0.05 vs. AcJun ^c^ *p* < 0.05 vs. AcJul; ^d^ *p* < 0.05 vs. AcAug.

## Data Availability

The data presented in this article are available on request from the corresponding authors.

## Sample Availability

Samples used are available from the authors.
